# SGCAST: symmetric graph convolutional auto-encoder for scalable and accurate study of spatial transcriptomics

**DOI:** 10.1093/bib/bbad490

**Published:** 2024-01-03

**Authors:** Jinzhao Li, Jiong Wang, Zhixiang Lin

**Affiliations:** Department of Statistics, The Chinese University of Hong Kong, Sha Tin, Hong Kong, China; School of Science and Engineering, The Chinese University of Hong Kong (Shenzhen), Shenzhen, 518172, China; Department of Statistics, The Chinese University of Hong Kong, Sha Tin, Hong Kong, China

**Keywords:** spatial transcriptomics data, spatial domain identification, symmetric graph convolutional auto-encoder, mini-batch training strategy

## Abstract

Recent advances in spatial transcriptomics (ST) have enabled comprehensive profiling of gene expression with spatial information in the context of the tissue microenvironment. However, with the improvements in the resolution and scale of ST data, deciphering spatial domains precisely while ensuring efficiency and scalability is still challenging. Here, we develop SGCAST, an efficient auto-encoder framework to identify spatial domains. SGCAST adopts a symmetric graph convolutional auto-encoder to learn aggregated latent embeddings via integrating the gene expression similarity and the proximity of the spatial spots. This framework in SGCAST enables a mini-batch training strategy, which makes SGCAST memory-efficient and scalable to high-resolution spatial transcriptomic data with a large number of spots. SGCAST improves the overall accuracy of spatial domain identification on benchmarking data. We also validated the performance of SGCAST on ST datasets at various scales across multiple platforms. Our study illustrates the superior capacity of SGCAST on analyzing spatial transcriptomic data.

## INTRODUCTION

In biology, the spatial concept is important as it allows us to describe interactive biological networks, where each element is influenced by its surrounding environment [1]. In particular, the coordinated gene expression in tissues can be used to uncover its functions and connections [2]. Captured locations in spatial transcriptomics (ST) data are known as ‘spots’. However, early techniques such as 10X Visium [3] have limited resolution that cannot reach the cellular level. Each spot of 10X Visium is 55 $\mu $m in diameter and may contain tens of cells, and the number of spots on a tissue slide is at most 5000. Recently, there has been development in high-resolution ST data, such as Seq-scope [4] and Stereo-seq [5], that enables the profiling of gene expression at cellular/subcellular resolution. These methods generate datasets of a much larger scale, where the number of spots can exceed 100k [5]. Identification of spatial domains (regions characterized by similar expression patterns in space) has been one of the most important tasks in ST. With the explosion in the scale of ST data, scalable methods that can be implemented on high-resolution ST data are in great demand.

Methods have been developed for dimension reduction and clustering of scRNA-Seq data, such as scVI [6] and ZIFA [7]. Although these methods can be implemented on the spot by gene matrix in ST data in principle, they may lose information and efficiency because they ignore the spatial information of the spots. On the other hand, methods have been developed for ST data where the information on the spatial location of the spots is incorporated. BayesSpace [8] employs a fully Bayesian method that encourages the clustering of nearby locations through a prior that stores neighborhood information. stLearn [9] employed integrative analysis to use all information including histology image, gene expression and spatial coordinates, first finding cell types, then reconstructing cell types in a tissue and finally deciphering tissue regions with high cell-to-cell interactions. SpaGCN [10] utilizes a graph convolutional network to incorporate information from gene expression, histological image and spatial coordinates, coupled with a self-supervised module to train the neural network. SEDR [11] uses a deep auto-encoder to create a low-dimensional latent representation of gene expression, which is then merged with the corresponding spatial embedding obtained from a variational graph auto-encoder. SpatialPCA [12] extends the probabilistic version of principal component analysis (PCA) by incorporating location information and explicitly representing the spatial correlation structure through a kernel matrix. BASS [13] performs cell type clustering and spatial domain detection simultaneously within a Bayesian hierarchical modeling framework. DeepST [14] uses multiple neural networks to extract information from tissue morphology and spatial location, then combines them with gene expression to generate the representation of spots. STAGATE [15] generates low-dimensional latent embeddings via a graph attention auto-encoder, which adaptively aggregates information from its neighbors. Although these methods incorporate spatial information in analyzing ST data, they rely on a graph constructed from all spots in a sample. This makes them difficult to handle high-resolution ST data where the size of the graph is large because of the large number of spots. Mini-batch training may be a remedy, where a subset of the spots is used in each iteration of the update. However, it is challenging to implement mini-batch training for the setting of spatial transcriptomic data, because of the relationship of the spots in the graph. Therefore, there is a need for scalable methods in analyzing high-resolution spatial transcriptomic data with a large number of spots.

To address the need, we develop SGCAST, which is based on a symmetric graph convolutional auto-encoder for the analysis of Spatially resolved Transcriptomic data. SGCAST utilizes an auto-encoder for the graph convolution layer, where it employs the idea of Laplacian smoothing and Laplacian sharpening to construct its encoder and decoder. The auto-encoder in SGCAST incorporates the information of both the gene expression similarity and the proximity of the spatial spots, which effectively borrows information across the dataset and enables accurate identification of the spatial domains. This framework in SGCAST enables mini-batch training, where the spots in ST data are processed in a series of mini-batches. Instead of constructing a large graph with all the spots, which can be computationally intensive, individual small graphs are constructed using the spots in each batch. This mini-batch training strategy can significantly reduce computational time and memory usage for large-scale ST data. We demonstrate the superior performance of SGCAST in multiple experimental platforms, including 10X Visium [3], Seq-scope [4] and Stereo-seq [5]. The embeddings obtained by SGCAST can be used for spatial clustering mainly. In addition, the detected clusters can be effectively leveraged for downstream analysis, including differentially expressed genes detection and trajectory inference.

## RESULTS

### SGCAST improves the accuracy of identifying the layers in human dorsolateral prefrontal cortex

To quantitatively evaluate the performance of SGCAST on spatial clustering, we initiated applying it to the benchmarking dataset, which is a publicly available 10X Visium dataset including 12 slides of the human dorsolateral prefrontal cortex (DLPFC). The layers and white matter (WM) are annotated by Maynard *et al*. [16] based on the cytoarchitecture and gene markers (Figure 2A). Regarding it as the label, we compared SGCAST with several existing spatial clustering approaches on the accuracy (stLearn [9], BayesSpace [8], SpaGCN [10], SEDR [11], SpatialPCA [12], BASS [13], DeepST [14] and STAGATE [15]). We used adjusted rand index (ARI) [17] as the criterion, and the results demonstrated that our proposed SGCAST identifies the cortical layers effectively and outperforms other methods in 12 slides of DLPFC (Figure 2C, Supplementary Figures 1, 2 and 3). In the DLPFC slide 151673, SGCAST can detect all the layers and achieve the highest accuracy in clustering (ARI = 0.60) (Figure 2B). SpatialPCA, DeepST and STAGATE also performed well (ARI=0.58), but they did not detect layer 2 or layer 4 and separated WM into two clusters. Both BayesSpace and BASS did not detect layer 4, and they also separated WM into two clusters. To verify the effectiveness of the two graph convolutional layers in SGCAST, we implemented simplified versions of SGCAST, with only the layer capturing gene expression similarity, SGCAST$_{\textrm{exp}}$ or the layer capturing spatial proximity, SGCAST$_{\textrm{spa}}$. Results in Figure 2C demonstrate that both layers are important and the full version of SGCAST outperforms the simplified versions with either layer.

**Figure 1:**
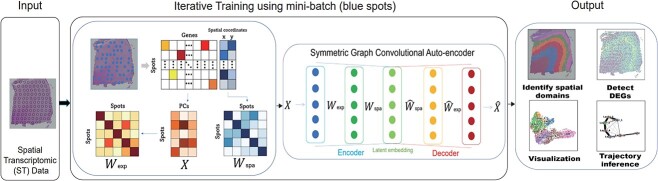
Overview of SGCAST. SGCAST takes spatial coordinates and gene expression of the spots in spatial transcriptomic data as input. The PCs are first computed from the gene expression of all the spots. SGCAST implements a symmetric graph convolutional auto-encoder with mini-batch training, which makes it scalable to large datasets. The auto-encoder takes the PCs of gene expression, adjacency matrix $W_{exp}$ representing gene expression similarity and adjacency matrix $W_{spa}$ representing spatial proximity of the spots, and outputs the latent embeddings of the spots. The embeddings can be used for clustering and visualization. Additionally, the detected clusters are used as the basis for further downstream analysis, including DEG detection and trajectory inference. The main usage of SGCAST is to provide an efficient and accurate spatial domain identification method that can be used as a basis for further downstream analysis.

**Figure 2:**
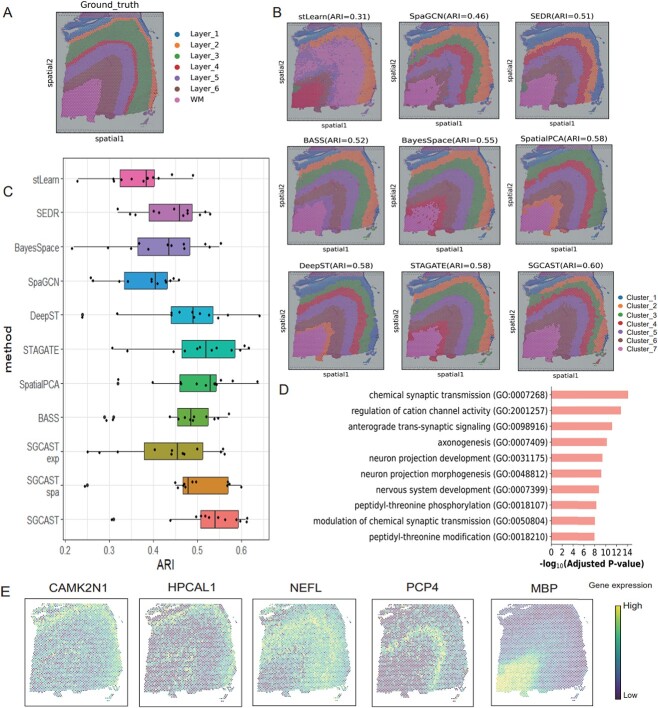
SGCAST displays accurate detection of spatial domains in the DLPFC datasets. **A** Ground truth of the layer structure in slide 151673 [16]. **B** Clustering assignments generated by stLearn, SpaGCN, SEDR, BASS, BayesSpace, SpatialPCA, DeepST, STAGATE and SGCAST in slide 151673. **C** Boxplot displaying the clustering accuracy in all 12 slides of the DLPFC dataset, measured in terms of ARI scores, for 11 different methods. The median, upper and lower quartiles, and 1.5$\times $ interquartile range in the boxplot are represented by the center line, box limits and whiskers, respectively. **D** The enrichment analysis of GO for differentially expressed genes (161 genes) in domain 2 (orange layer in the result). **E** Visualization of gene expression for layer-specific genes in slide 151673.

We also performed differential expression analysis to verify the biological meaning of the domains identified from SGCAST. Specifically, we use the detected clusters as the basis to find differentially expressed genes in each spatial domain. The detected differentially expressed genes (DEGs) display clear expression patterns (Figure 2E). Among these genes, PCP4 is a known marker gene in the prefrontal cortex [18]. An additional Gene Ontology (GO) analysis was performed on the detected DEGs specific to cluster 2 (orange) with *P* value less than 0.01 (Figure 2D). The enriched GO terms include chemical synaptic transmission, regulation of cation channel activity and anterograde trans-synaptic signaling, which is consistent with the function of the marker gene for cluster 2: CAMK2N1 is differentially expressed in cluster 2 and it was shown to regulate long-term synaptic activity [19]. In addition, another marker gene for cluster 2, HPCAL1, promotes glioblastoma proliferation, which is a prevalent primary cancer with evident aggressiveness in the human brain [20].

### SGCAST exhibits a layered pattern clearly mirroring the layers in the annotation of the mouse olfactory bulb

**Figure 3:**
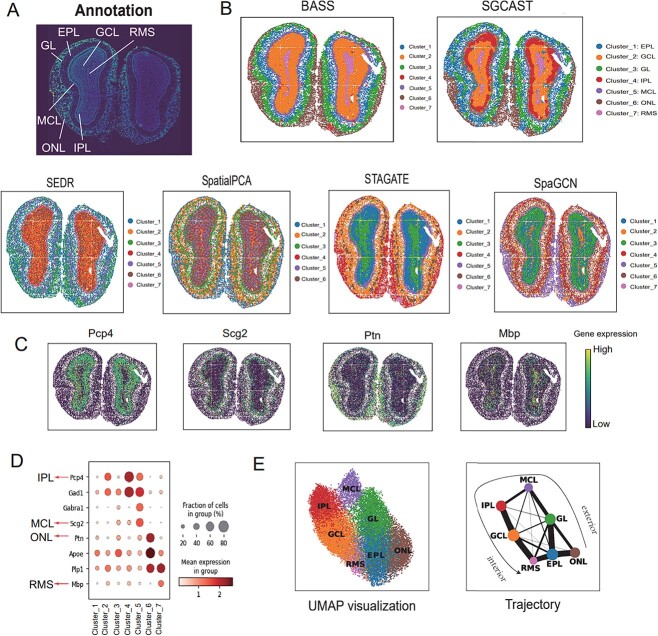
SGCAST detects finer layer structure in the mouse olfactory bulb. **A** The DAPI-stained image with annotation for Stereo-seq mouse olfactory bulb [11]. **B** Spatial domains identified by BASS, SEDR, SpatialPCA, STAGATE, SpaGCN (without histology image) and SGCAST in the Stereo-seq mouse olfactory bulb. **C** Visualization of gene expression for the detected marker genes. **D** Dotplot of visualization of expression fraction for layer-specific genes. **E** Results of UMAP and PAGA, generated using the SGCAST embeddings.

Next, we applied SGCAST to the dataset of the mouse olfactory bulb generated by Stereo-seq [5]. Stereo-seq combines DNA nanoball-patterned arrays and *in situ* RNA capture and creates spatial transcriptomic data with subcellular resolution. The spots in this dataset were binned into a resolution of around $14 \mu $m [5]. SGCAST was able to accurately identify the laminar organization of various layers in the mouse olfactory bulb, including the rostral external plexiform layer (EPL), granule cell layer (GCL), glomerular layer (GL), internal plexiform layer (IPL), mitral cell layer (MCL), olfactory nerve layer (ONL) and rostral migratory stream (RMS), which matches the known anatomical characteristics seen in the DAPI-stained image (Figure 3A). In comparison with other methods such as SpaGCN, SEDR and SpatialPCA, SGCAST identified a much finer layered structure (Figure 3B). Although STAGATE and BASS also identified a clear pattern in general, STAGATE did not separate the RMS and the GCL, while BASS did not distinguish between the IPL and the GCL. BayesSpace and stLearn were not implemented for this dataset due to the high memory cost and DeepST was not implemented because it requires the histology image.

We further analyzed DEGs among domains identified by SGCAST (Figure 3C, D). By calculating the top genes, our analysis revealed genes that display predominant expression in specific layers of the olfactory bulb tissue: Pcp4 is predominantly expressed in the IPL, Ptn in the ONL, Scg2 in the MCL and Mbp in the RMS (Figure 3C, D), which are consistent with the maker genes reported in mouse olfactory bulb [21].

Finally, based on the spatial domains identified by SGCAST (Figure 3E), the UMAP visualization and trajectory inference by PAGA [22] denoted that the olfactory bulb layers revealed a clear organization from the exterior of the mouse olfactory bulb to the interior, following the spatial distribution in the annotation.

### SGCAST precisely identifies tissue structures in the mouse colon from Seq-scope

**Figure 4:**
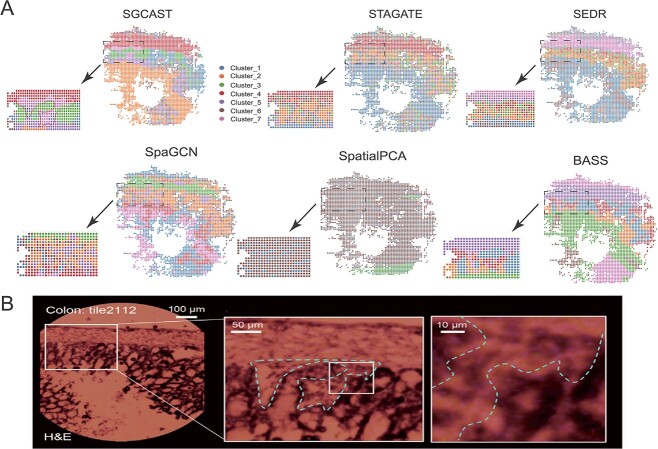
SGCAST can decipher complex tissue structures in the mouse colon. **A** Spatial domains identified by SGCAST, STAGATE, SEDR, SpaGCN (without histology image), SpatialPCA and BASS in the Seq-scope mouse colon tissue section. **B** Underlying H&E histology of the mouse colon [4].

In addition to the 10X Genomics Visium platform and Stereo-seq platform, we verified the effectiveness of SGCAST on a Seq-scope dataset with the spatial resolution of 10 $\mu $m generated from mouse colon [4]. When compared with the 55 $\mu $m resolution of the 10X Visium platform, Seq-scope enables the profiling of spatial expressions at the cellular level with a larger number of spots and the pixels in Seq-Scope are around 0.5–0.8 $\mu $m apart from each other. As a gastrointestinal organ, the colon consists of complex tissue layers with histological zonation structure and diverse cellular components [23]. From a histological perspective, the colonic wall can be divided into the colonic mucosa and the external muscle layers [24]. Within the colonic wall, the colonic mucosa comprises the epithelium and lamina propria. The epithelium can be further subdivided into the crypt-base, transitional and surface layers [4]. Annotation of the gridded Seq-Scope dataset (Supplementary Figure 4) revealed transcriptome phenotypes corresponding to these layers. SGCAST effectively uncovers the tissue structures in the mouse colon tissue, and it accurately deciphered the region of the lamina propria, highlighted by the red solid line in Figure 4A. This region is supported by the histology image (blue dashed curve) in Figure 4B. The clustering result given by SGCAST is close to the annotated layers (Supplementary Figure 4). BASS and STAGATE also performed well, but they were not able to clearly recover the region of lamina propria (Figure 4A). SEDR and SpaGCN did not capture the layered pattern as well, and they also did not recover the region of lamina propria (Figure 4A). SpatialPCA did not work well on this dataset.

### SGCAST is scalable and efficient for large-scale ST data

**Figure 5:**
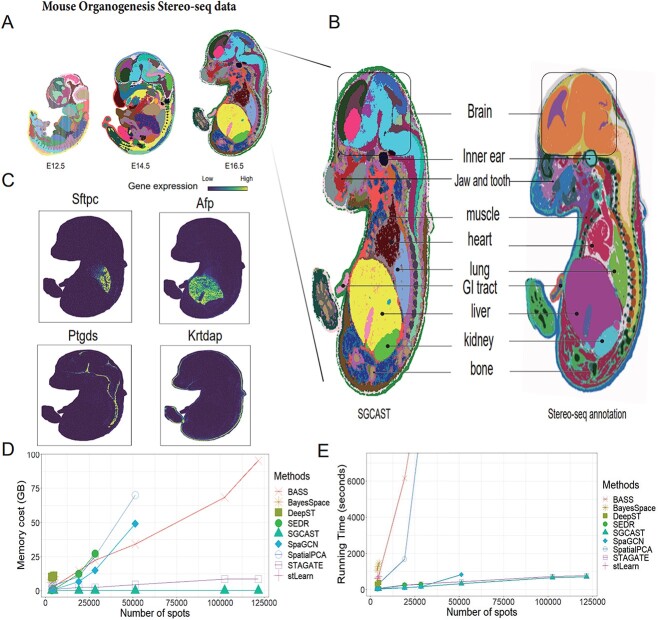
SGCAST works on large-scale ST data accurately and efficiently. **A** Spatial clustering by SGCAST on mouse embryo E12.5, E14.5 and E16.5 days. **B** Comparison of spatial domains of major tissues identified by SGCAST with Stereo-seq annotation on mouse embryo E16.5 days [5]. **C** Visualization of gene expression for the detected domain-specific marker genes. **D** Comparison of memory cost in the real datasets by stLearn, BayesSpace, SpaGCN, SEDR, BASS, SpatialPCA, DeepST, STAGATE and SGCAST. **E** Comparison of running time in the real datasets by stLearn, BayesSpace, SpaGCN, SEDR, BASS, SpatialPCA, DeepST, STAGATE and SGCAST.

With advances in technology, some ST methods can measure a large number of cells through high spatial resolutions on a great scale. For instance, Stereo-seq launched the MOSTA, a mouse organogenesis spatiotemporal transcriptomic atlas aimed at mapping the spatiotemporal transcriptomic dynamics of the whole developing mouse embryo [5]. Here, we applied SGCAST to three late embryonic stages, including E12.5, E14.5 and E16.5 days (Figure 5A). The numbers of spots are 51k, 102k and 121k, respectively. The mini-batch training strategy employed by SGCAST allows it to be scalable and efficient, even for massive spatial transcriptomic datasets. In contrast, other spatial clustering methods such as SEDR failed due to their high memory cost, while BASS and SpatialPCA failed due to their long-running time, which can take more than a day to complete. This highlights the advantage of SGCAST’s implementation for large-scale spatial transcriptomic data analysis.

We evaluated the performance of SGCAST in section E16.5. The comparison (Figure 5B) exhibits that SGCAST accurately identifies most tissues, including nervous system, inner ear, muscle, heart, lung, GI tract, liver, kidney and bone, which matched the manually annotated areas provided by Stereo-seq confirmed by visualizing specific marker genes [5]. Moreover, we analyzed DEGs between the spatial domains identified by SGCAST and discovered canonical marker genes for the organs: Ptgds [25] in meninges, Krtdap [26] in the epidermis, Sftpc [27] in the lung, Afp [28] in the liver (Figure 5C). These results collectively demonstrate the scalability of SGCAST in identifying tissue structures from massive spatial transcriptomic data.

In addition to the evaluation of clustering performance, we also recorded the memory usage and running time of SGCAST and other methods on real datasets (Figure 5D, E). Specifically, we implemented these methods on spatial transcriptomic data with the number of spots ranging from 3k to 121k (Supplementary Table 1). Compared with the other methods, SGCAST is highly memory-efficient due to its mini-batch training, requiring only around 0.25GB of GPU memory to implement on datasets with various scales (Figure 5D). The memory usage for the other methods is either linear or quadratic in the number of spots, which limits their usage in high-resolution spatial transcriptomic data with a large number of spots. On the contrary, the memory usage of SGCAST does not depend on the total number of spots and mainly depends on the size of the mini-batch. Similar to SGCAST, SpaGCN is also based on a graph convolutional network. However, it is less memory-efficient compared with SGCAST. Because SpaGCN relies on the neighboring graph constructed from all the spots, it is difficult to implement mini-batch training. The running time was capped at 2 h in Figure 5E and the running time for methods that cannot be implemented due to high memory usage is not shown. For all datasets, it took SGCAST less than 20 min to implement, significantly faster than the other methods. The running time not displayed in Figure 5E is as follows: for the mouse Seq-scope colon dataset (28 399 spots), it took BASS and SpatialPCA 3.2 and 2.5 h, respectively; for the Stereo-seq mouse embryo E12.5 dataset (51 335 spots), it took BASS and SpatialPCA 8.1 and 5.1 h, respectively; for the Stereo-seq mouse embryo E14.5 (102 489 spots) and E16.5 (121 764 spots) datasets, it took BASS and SpatialPCA more than 12 h to implement. To summarize, SGCAST is both memory-efficient and computationally fast to be implemented on high-resolution spatial transcriptomic data with a large number of spots.

## DISCUSSION

With the rapid advances in ST technology, there is an inevitable trend toward higher spatial resolution and larger data scales. In this paper, we present SGCAST, a simple and efficient framework for identifying spatial domains. Firstly, SGCAST uses an auto-encoder structure where the encoder aggregates information to perform smoothing, while the decoder separates the information to perform sharpening. Secondly, SGCAST employs information efficiently by transforming information from gene expression and the position of spots into two adjacency matrices and further integrating them using the auto-encoder. Lastly, SGCAST utilizes the mini-batch training strategy, avoiding intermediate clustering throughout the training process, and displaying superior efficiency and scalability to large ST datasets. These factors contribute to the superior performance of SGCAST, which not only accurately identifies spatial domains but also extracts spatially variable genes crucial for revealing tissue layout and inferring biological purposes.

We demonstrated the superior performance of SGCAST on multiple ST datasets from different platforms with various spatial resolutions. SGCAST precisely revealed the layer organization of the human DLPFC from 10X Visium and the mouse olfactory bulb from Stereo-seq, and facilitates the detection of differentially expressed genes over identified domains. On average, SGCAST achieved the highest ARI compared with other spatial clustering methods, indicating that the spatial domains identified by SGCAST are more biologically meaningful. We also found that SGCAST accurately identified the complex tissue structures in the mouse colon. Additionally, we demonstrated the scalability of SGCAST on the Embryo dataset and showed that SGCAST identified the major tissue structures of Embryo E16.5 clearly. Finally, we compared the efficiency of SGCAST with other popular methods in both running time and memory cost and found that SGCAST was highly efficient.

We note that SpaGCN [10] also employs graph convolutional networks. However, there is a primary difference between SpaGCN and SGCAST: SpaGCN implements the unsupervised deep embedding (UDE) framework [29], while SGCAST implements the symmetric graph convolutional auto-encoder [30]. It is challenging to implement mini-batch training in UDE for the setting of ST data: UDE requires the search of cluster centroids and assigning cluster labels to the spots in the iterations; however, the cluster centroids for different batches are likely different and it is difficult to align these centroids across batches. On the contrary, the auto-encoder framework in SGCAST is more adaptable for mini-batch training. In addition, we also notice that SEDR [11] employs two auto-encoders with graph convolutional layers. However, the structure and intuition of the auto-encoder are fundamentally different. SEDR utilizes a variational graph auto-encoder to encode spatial information and a deep auto-encoder to embed transcriptional expression and concatenates the learned features. In contrast, SGCAST uses a symmetric graph convolutional auto-encoder to aggregate the information, rather than concatenating it. This approach allows SGCAST to better capture the spatial information and improve the clustering performance. (More details are listed in Supplementary materials.) The input of SGCAST is the top principal components (PCs) and PCs go through the symmetric graph convolutional auto-encoder to learn the latent embedding. To display the significance of the auto-encoder, we compared results obtained by applying mclust directly to PCs with those using latent embeddings learned by the auto-encoder on the DLPFC dataset. The comparison demonstrates that the clustering result using the latent embeddings by SGCAST is much better compared with using directly the PCs (Supplementary Figure 5), which means the auto-encoder plays a critical role in aggregating information. SGCAST uses the PCs as input, which may lead to some loss of information, compared with using the whole transcriptome. However, using PCs benefits noise reduction and increases the signal-to-noise ratio in the data, compared with using the whole transcriptome. Choosing the number of PCs is a balance of signal-to-noise: more PCs preserve more information but can also include more noise, while fewer PCs may contain less noise but lose information. We found that the top 50 PCs strike a good balance of signal-to-noise, and we use the same number of top PCs for all the datasets, including the DLPFC dataset, Stereo-seq mouse olfactory bulb and embryo and Seq-scope mouse colon. In our experiments, the number of PCs has a larger impact on the human DLPFC datasets, and the results for Stereo-seq mouse olfactory bulb and Seq-scope mouse colon datasets are more robust to the number of PCs (Supplementary Figures 6, 7 and 8). In addition, we ran multiple experiments regarding different training parameters and found all results of SGCAST are robust to these parameters (Supplementary Figures 9 to 14). For now, the double graph convolutional layers in SGCAST capture gene expression similarity and spatial proximity. One future direction is to further incorporate the information of histological images through an extra convolutional layer in SGCAST. Although we implemented SGCAST on spatial transcriptomic datasets, it would be interesting to test the performance of SGCAST on the emerging spatial epigenomic datasets [31–33].

In conclusion, SGCAST is an efficient and promising framework for learning integrated latent embeddings to decipher the spatial domain. With the advent of new ST technologies, we anticipate that SGCAST can assist in uncovering new biological insights in the spatial context.

## METHODS

### Overview of SGCAST

The core of SGCAST is a symmetric graph convolutional auto-encoder, which learns latent embeddings from spatial transcriptomic data. The symmetric graph convolutional auto-encoder combines the information of gene expression similarity and spatial proximity, through multi-view graph convolution layers, to effectively learn the latent embeddings of the spatial spots. One important feature of SGCAST is the mini-batch training strategy, which makes SGCAST memory-efficient and scalable. The latent embeddings given by SGCAST can be used for clustering, data visualization, trajectory inference and other downstream analyses. A graphical overview of SGCAST is shown in Figure 1.

### Symmetric graphical auto-encoder

SGCAST first runs PCA on the preprocessed gene expression matrix of all spots and the top 50 PCs are used as the input of the symmetric graphical auto-encoder.

#### Mini-Batch training strategy

Previous methods [9–11] employing spatial information generally build a spatial network for the whole data to describe the neighborhood before training the model, which may be memory-consuming and difficult to implement on large spatial transcriptomic data with high resolution. Unlike these methods, SGCAST regards each spot in the spatial transcriptomic data as a data point and uses a mini-batch training strategy to train the parameters in graph convolutional layers. SGCAST generates adjacency matrices for the mini-batch picked in each iteration. Suppose the total number of spots and the size of the mini-batch are N and n, respectively, then the number of iterations in each epoch equals $\lceil N/n \rceil $. ($n$ is set as 2000 by default.)

#### Multi-view graph convolutional layers

SGCAST aggregates information among the spots in each mini-batch by two factors: (1) the gene expression similarity of the spots, and (2) the physical proximity of the spots in the tissue slide. This is accomplished by constructing two graph convolutional layers in the encoder: one layer incorporates the gene expression similarity of the spots, and the other one incorporates the spatial proximity of the spots. To achieve this, SGCAST builds an adjacency matrix for each layer where the entries in the matrix measure the relatedness between spots in a mini-batch and are negatively associated with their distances [10]. For the layer that captures the gene expression similarity, the distance is calculated as 


(1) \begin{align*}& d_{e}(u,v)=\|\vec{x}_{u}-\vec{x}_{v}\|_{2},\end{align*}

where $\vec{x}_{u}$ and $\vec{x}_{v}$ are vectors of the PCs for spots $u$ and $v$, respectively. For the layer that captures the spatial proximity, the distance is calculated as 


(2) \begin{align*}& d_{p}(u,v)=\|\vec{p}_{u}-\vec{p}_{v}\|_{2},\end{align*}

where $\vec{p}_{u}$ and $\vec{p}_{v}$ are spatial coordinates for spots $u$ and $v$. The entries in the adjacency matrices $W_{e} = [w_{e} (u, v)]$ and $W_{p} = [w_{p} (u, v)]$ are then computed as 


(3) \begin{align*}& w_{e} (u, v)=\exp\left(-\frac{d_{e}(u,v)^{2}}{2l_{e}^{2}}\right),\end{align*}

and 


(4) \begin{align*}& w_{p} (u, v)=\exp\left(-\frac{d_{p}(u,v)^{2}}{2l_{p}^{2}}\right),\end{align*}

where $l_{e}$ and $l_{p}$ influence how quickly the weight decays as a function of distance, and are dynamically determined for each mini-batch in SGCAST. After a mini-batch is input into the framework, distances are calculated using equations 1 and 2, and the $\tau _{e}$th-quantile of the distance $d_{e}$ and the $\tau _{p}$th-quantile of the distance $d_{p}$ are determined using *torch.quantile()*. The values of $l_{e}$ and $l_{p}$ are then computed by solving equations where $-\frac{d_{e\tau _{e}}^{2}}{2l_{e}^{2}}=m$ and $-\frac{d_{p\tau _{p}}^{2}}{2l_{p}^{2}}=m$, where $m$ is the largest integer such that $torch.exp(m)=0$. This ensures that the proportions of non-zero entries in the non-diagonal elements of the adjacency matrices $W_{e}$ and $W_{p}$ equal $\tau _{e}$ and $\tau _{p}$, respectively. The default values of $\tau _{e}$ and $\tau _{p}$ are set to 0.07. The reason for this rule is to ensure that the average number of neighbors for the spots in each mini-batch is the same.

#### Encoder

The encoder in SGCAST takes the top 50 PCs as the input and aggregates information to the embedding according to the adjacency matrices. Let $X_{i}$ be the PCs for the $i$-th mini-batch and the graph convolutional layer can be written as 


(5) \begin{align*}& H_{i}^{(k)}=\delta(W_{i}^{(k)}H_{i}^{(k-1)}B^{(k)}),\end{align*}

where $H_{i}^{(k)}$ is the embedding of mini-batch $i$ generated in layer $k$, (i.e. $H_{i}^{(0)}=X_{i}$), $W_{i}^{(k)}$ is the adjacency matrix for mini-batch $i$ in layer $k$, $B^{(k)}$ is a 50$\times $50 matrix representing the projection parameters of the $k$-th convolutional layer and $\delta (\cdot )$ is the nonlinear activation function. There are two convolutional layers (i.e. k $\in \{1, 2\}$): the first layer aggregates information based on gene expression similarity, and the second layer aggregates information based on spatial proximity. The output embedding of the encoder is the representation of the spots used for spatial domain identification.

#### Decoder

The reconstruction of node features in the decoder is designed based on Laplacian sharpening as the counterpart of Laplacian smoothing in the encoder (Supplementary note): the adjacency matrix $\hat{W}_{i}$ in the layer of the decoder has the form $3*I_{n}-W_{i}$, where $W_{i}$ is the adjacency matrix in the encoder [30, 34, 35]. $H_{i}^{(2)}$ is the result of the encoder, and it serves as the input for the decoder, i.e. $\hat{H}_{i}^{(2)}=H_{i}^{(2)}$. The $k$-th layer in the decoder reconstructs the embedding in layer $k-1$ as follows: 


(6) \begin{align*}& \hat{H}_{i}^{(k-1)}=\delta\Big(\hat{W}_{i}^{(k)}\hat{H}_{i}^{(k)}\hat{B}_{i}^{(k)}\Big),\end{align*}

where $\hat{W}_{i}^{(k)}=3*I_{n}-W_{i}^{(k)}$. The final output of the decoder, $\hat{H}^{(0)}$, is the reconstructed PCs. To avoid overfitting, SGCAST assumes symmetry in the encoder and decoder by setting $\hat{B}_{i}^{(k)} = (B_{i}^{(k)})^{T}$ for $k \in \{1, 2\}$.

#### Loss function and training details

The objective of SGCAST is to minimize the reconstruction loss of PCs as follows: 


(7) \begin{align*}& ||X_{i}-\hat{H}_{i}^{0}||_{2}.\end{align*}

SGCAST implements the SGD optimizer [36] to minimize the reconstruction loss. ELU [37] serves as the activation function. The number of iterations is set as 100 by default. The initial learning rate is set as 2e-1 and decreases to 1e-1 at the last 20 epochs.

### Data description

SGCAST is applicable to multiple ST datasets obtained from various platforms, such as 10X Visium, Seq-Scope and Stereo-seq. In DLPFC datasets, 12 tissue slides are annotated with DLPFC layers and WM regions with a spot range of 3498 to 4789 [16]. The Seq-Scope processed datasets for mouse colon are grid sampled at a spatial resolution of 10 $\mu $m with the number of spots equal to 28 399 [4]. The mouse olfactory bulb data from Stereo-seq consist of 19 109 spots with a resolution of around 14 $\mu $m. The Stereo-seq embryo data from E12.5 to E16.5 has been binned into spots with a diameter of 25 $\mu $m and the number of spots ranges from 51 335 to 121 764 [5].

### Data preprocessing

ST data used by SGCAST consist of a gene expression count matrix and a two-dimensional coordinates matrix for spots. First, we log-transform and normalize the raw gene expression by library size using Scanpy [38]. Next, we select the top 3000 highly variable genes and run PCA to the selected gene expression matrix. Finally, the top 50 PCs are used as inputs, which is effective across all datasets in the paper.

### Clustering and refinement

Different strategies are applied to perform spatial clustering. When the number of spatial domains is provided, SGCAST implements the mclust clustering algorithm [39] on the latent embeddings to obtain cluster labels for the spots. When there is a lack of prior information, the Louvain algorithm [40] is utilized for clustering. By default, the algorithm uses a resolution parameter of 1.2. Louvain algorithm builds a graph used for clustering. By adjusting the number of neighbors in the graph, the number of resulting clusters can be determined. After clustering, SGCAST provides a step to refine the clustering result. During this step, SGCAST evaluates the domain assignments for each spot and its surrounding spots. If over half of the spots surrounding a given spot are assigned to a different domain, that spot will be relabeled to match the major label of its surrounding spots. We performed cluster refinement for all ST datasets.

### Spatial trajectory inference

PAGA algorithm [22] is used to generate trajectory results.

### Identifying differentially expressed genes

Wilcoxon test in Scanpy [38] is employed to discover DEGs for each identified spatial domain (Benjamin–Hochberg adjustment).

### Gene Ontology enrichment analysis

For the DFPLC dataset, we conducted the gene set enrichment analysis implemented in GSEAPY package [41] to discover the enriched GO terms for spatially variable genes in the detected domain with adjusted *P* value < 0.01.

**Key Points**

- SGCAST adopts a symmetric graph convolutional auto-encoder to learn aggregated latent embeddings via integrating the gene expression similarity and the proximity of the spatial spots.
- For spatial domain identification, SGCAST consistently outperformed baseline methods on ST datasets at various scales across multiple platforms.
- SGCAST enables mini-batch training strategy, which makes it scalable to high-resolution spatial transcriptomic data.
- SGCAST facilitates the downstream analysis in the biological study, such as the detection of differentially expressed genes.

## Supplementary Material

Supplementary_file_bbad490

## Data Availability

All data utilized in the paper are accessible for open download. The DLPFC [16] is contained by spatialLIBD (http://spatial.libd.org/spatialLIBD/). The Seq-scope colon dataset is collected from the Seq-scope processed data website (https://deepblue.lib.umich.edu/data/concern/data_sets/9c67wn05f). The mouse olfactory bulb data generated by Stereo-seq data can be found at (https://github.com/JinmiaoChenLab/SEDR_analyses). The raw data of the Stereo-seq mouse embryo are downloaded from (https://db.cngb.org/stomics/mosta/download/) and the SAW pipeline (https://github.com/BGIResearch/SAW) is required to process the raw data.

## CODE AVAILABILITY

The SGCAST algorithm is implemented in Python and is freely accessible on GitHub (https://github.com/cuhklinlab/SGCAST).

## Author Biographies

**Jinzhao Li** is a PhD student in Department of Statistics in the Chinese University of Hong Kong.

**Jiong Wang** is an MPhil student in the School of Science and Engineering in the Chinese University of Hong Kong (Shenzhen).

**Zhixiang Lin** is an assistant professor in Department of Statistics in the Chinese University of Hong Kong.

## References

[1] Asp M, Bergenstråhle J, Lundeberg J. Spatially resolved transcriptomes—next generation tools for tissue exploration. Bioessays 2020;42(10):e1900221. 32363691 10.1002/bies.201900221

[2] Armingol E, Officer A, Harismendy O, Lewis NE. Deciphering cell–cell interactions and communication from gene expression. Nat Rev Genet 2021;22(2):71–88. 33168968 10.1038/s41576-020-00292-x PMC7649713

[3] Ji AL, Rubin AJ, Thrane K, et al. Multimodal analysis of composition and spatial architecture in human squamous cell carcinoma. Cell 2020;182(2):497–514.e22. 32579974 10.1016/j.cell.2020.05.039 PMC7391009

[4] Cho CS, Xi J, Si Y, et al. Microscopic examination of spatial transcriptome using Seq-scope. Cell 2021;184(13):3559–3572.e22. 34115981 10.1016/j.cell.2021.05.010 PMC8238917

[5] Chen A, Liao S, Cheng M, et al. Spatiotemporal transcriptomic atlas of mouse organogenesis using DNA nanoball-patterned arrays. Cell 2022;185(10):1777–1792.e21. 35512705 10.1016/j.cell.2022.04.003

[6] Lopez R, Regier J, Cole MB, et al. Deep generative modeling for single-cell transcriptomics. Nat Methods 2018;15(12):1053–8. 30504886 10.1038/s41592-018-0229-2 PMC6289068

[7] Pierson E, Yau C. ZIFA: dimensionality reduction for zero-inflated single-cell gene expression analysis. Genome Biol 2015;16(1):1–10. 26527291 10.1186/s13059-015-0805-z PMC4630968

[8] Zhao E, Stone MR, Ren X, et al. Spatial transcriptomics at subspot resolution with bayesspace. Nat Biotechnol 2021;39(11):1375–84. 34083791 10.1038/s41587-021-00935-2 PMC8763026

[9] Pham D, Tan X, Xu J, et al. stLearn: integrating spatial location, tissue morphology and gene expression to find cell types, cell-cell interactions and spatial trajectories within undissociated tissues bioRxiv preprint. 2020. 10.1101/2020.05.31.125658.

[10] Hu J, Li X, Coleman K, et al. SpaGCN: integrating gene expression, spatial location and histology to identify spatial domains and spatially variable genes by graph convolutional network. Nat Methods 2021;18(11):1342–51. 34711970 10.1038/s41592-021-01255-8

[11] Fu H, Xu H, Chong K, et al. Unsupervised spatially embedded deep representation of spatial Transcriptomics bioRxiv preprint. 2021. 10.1101/2021.06.15.448542. PMC10790257 38217035

[12] Shang L, Zhou X. Spatially aware dimension reduction for spatial transcriptomics. Nat Commun 2022;13(1):7203. 36418351 10.1038/s41467-022-34879-1 PMC9684472

[13] Li Z, Zhou X. BASS: multi-scale and multi-sample analysis enables accurate cell type clustering and spatial domain detection in spatial transcriptomic studies. Genome Biol 2022;23(1):168. 35927760 10.1186/s13059-022-02734-7 PMC9351148

[14] Xu C, Jin X, We S, et al. DeepST: identifying spatial domains in spatial transcriptomics by deep learning. Nucleic Acids Res 2022;50(22):e131–1. 36250636 10.1093/nar/gkac901 PMC9825193

[15] Dong K, Zhang S. Deciphering spatial domains from spatially resolved transcriptomics with an adaptive graph attention auto-encoder. Nat Commun 2022;13(1):1739. 35365632 10.1038/s41467-022-29439-6 PMC8976049

[16] Maynard KR, Collado-Torres L, Weber LM, et al. Transcriptome-scale spatial gene expression in the human dorsolateral prefrontal cortex. Nat Neurosci 2021;24(3):425–36. 33558695 10.1038/s41593-020-00787-0 PMC8095368

[17] Hubert L, Arabie P. Comparing partitions. J Classif 1985;2:193–218.

[18] Watakabe A, Hirokawa J, Ichinohe N, et al. Area-specific substratification of deep layer neurons in the rat cortex. J Comp Neurol 2012;520(16):3553–73. 22678985 10.1002/cne.23160

[19] Astudillo D, Karmelic D, Casas BS, et al. CaMKII inhibitor 1 (CaMK2N1) mRNA is upregulated following LTP induction in hippocampal slices. Synapse 2020;74(10):e22158. 32320502 10.1002/syn.22158 PMC8108577

[20] Zhang D, Liu X, Xu X, et al. HPCAL 1 promotes glioblastoma proliferation via activation of Wnt/*$\beta $*-catenin signalling pathway. J Cell Mol Med 2019;23(5):3108–17. 30843345 10.1111/jcmm.14083 PMC6484330

[21] Vickovic S, Eraslan G, Salmén F, et al. High-definition spatial transcriptomics for in situ tissue profiling. Nat Methods 2019;16(10):987–90. 31501547 10.1038/s41592-019-0548-y PMC6765407

[22] Wolf FA, Hamey FK, Plass M, et al. PAGA: graph abstraction reconciles clustering with trajectory inference through a topology preserving map of single cells. Genome Biol 2019;20:1–9. 30890159 10.1186/s13059-019-1663-x PMC6425583

[23] Levine DS, Haggitt RC. Normal histology of the colon. Am J Surg Pathol 1989;13(11):966–84. 2679155 10.1097/00000478-198911000-00008

[24] Farkas AE, Gerner-Smidt C, Lili L, et al. Cryosectioning method for microdissection of murine colonic mucosa. J Vis Exp 2015;101:e53112. 10.3791/53112 PMC4544980 26274554

[25] Harrington MG, Fonteh AN, Biringer RG, et al. Prostaglandin D synthase isoforms from cerebrospinal fluid vary with brain pathology. Dis Markers 2006;22(1–2):73–81. 16410653 10.1155/2006/241817 PMC3851407

[26] Bazzi H, Fantauzzo KA, Richardson GD, et al. Transcriptional profiling of developing mouse epidermis reveals novel patterns of coordinated gene expression. Dev Dyn 2007;236(4): 961–70. 17330888 10.1002/dvdy.21099

[27] Brasch F, Griese M, Tredano M, et al. Interstitial lung disease in a baby with a de novo mutation in the SFTPC gene. Eur Clin Respir J 2004;24(1):30–9. 10.1183/09031936.04.00000104 15293602

[28] Kuhlmann WD, Peschke P. Hepatic progenitor cells, stem cells, and AFP expression in models of liver injury. Int J Exp Pathol 2006;87(5):343–59. 16965562 10.1111/j.1365-2613.2006.00485.x PMC2517380

[29] Xie J, Girshick R, Farhadi A. Unsupervised deep embedding for clustering analysis. In International conference on machine learning, 2016; p. 478–487. PMLR.

[30] Park J, Lee M, Chang HJ, et al. Symmetric graph convolutional autoencoder for unsupervised graph representation learning. In Proceedings of the IEEE/CVF International Conference on Computer Vision, 2019; 6519–28.

[31] Deng Y, Bartosovic M, Ma S, et al. Spatial profiling of chromatin accessibility in mouse and human tissues. Nature 2022;609(7926):375–83. 35978191 10.1038/s41586-022-05094-1 PMC9452302

[32] Llorens-Bobadilla E, Zamboni M, Marklund M, et al. Solid-phase capture and profiling of open chromatin by spatial ATAC. Nat Biotechnol 2023;41:1085–8. 36604544 10.1038/s41587-022-01603-9 PMC10421738

[33] Deng Y, Bartosovic M, Kukanja P, et al. Spatial-CUT&tag: spatially resolved chromatin modification profiling at the cellular level. Science 2022;375(6581):681–6. 35143307 10.1126/science.abg7216 PMC7612972

[34] Kipf TN, Welling M. Semi-supervised classification with graph convolutional networks. In Proc. International Conference on Learning Representations, 2016.

[35] Li Q, Han Z, Wu XM. Deeper insights into graph convolutional networks for semi-supervised learning. In Thirty-Second AAAI conference on artificial intelligence, 2018.

[36] Bottou L. Large-scale machine learning with stochastic gradient descent. In Proceedings of COMPSTAT’2010: 19th International Conference on Computational StatisticsParis France, August 22–27, 2010 Keynote, Invited and Contributed Papers, 2010; p. 177–186.

[37] Clevert DA, Unterthiner T, Hochreiter S. Fast and accurate deep network learning by exponential linear units (elus) arXiv preprint arXiv:1511.07289. 2015.

[38] Wolf F, Angerer P, Theis FJ. SCANPY: large-scale single-cell gene expression data analysis. Genome Biol 2018;19:1–5. 29409532 10.1186/s13059-017-1382-0 PMC5802054

[39] Fraley C, Raftery AE, Murphy TB. Mclust version 4 for R: normal mixture modeling for model-based clustering, classification, and density estimation. R J 2012;8:289–317. PMC5096736 27818791

[40] Blondel VD, Guillaume JL, Lambiotte R, Lefebvre E. Fast unfolding of communities in large networks. J Stat Mech Theory Exp 2008;2008(10):P10008.

[41] Fang Z, Liu X, Peltz G. GSEApy: a comprehensive package for performing gene set enrichment analysis in python. Bioinformatics 2023;39(1):btac757. 36426870 10.1093/bioinformatics/btac757 PMC9805564

