# Supplementary material for: SGCAST: symmetric graph convolutional auto-encoder for scalable and accurate study of spatial transcriptomics
Source: Brief Bioinform. 2024 Jan 3;25(1):bbad490. doi: 10.1093/bib/bbad490 (PMC10782917; doi:10.1093/bib/bbad490)
Supplement: Supplementary_file_bbad490 [file supplementary_file_bbad490.docx]

**Supplementary Information for**

**SGCAST: Symmetric Graph Convolutional
Auto-encoder for scalable and accurate study of
Spatial Transcriptomics**

Jinzhao Li^1^, Jiong Wang^2^ and Zhixiang Lin^1,*,^

^1^Department of Statistics, The Chinese University of Hong Kong, Sha Tin, Hong Kong SAR, China

2School of Science and Engineering, The Chinese University of Hong Kong (Shenzhen), Shenzhen, 518172, China

^*^Corresponding author: Zhixiang Lin, Department of Statistics, The Chinese University of

Hong Kong, Sha Tin, Hong Kong SAR, China. Email: zhixianglin@cuhk.edu.hk


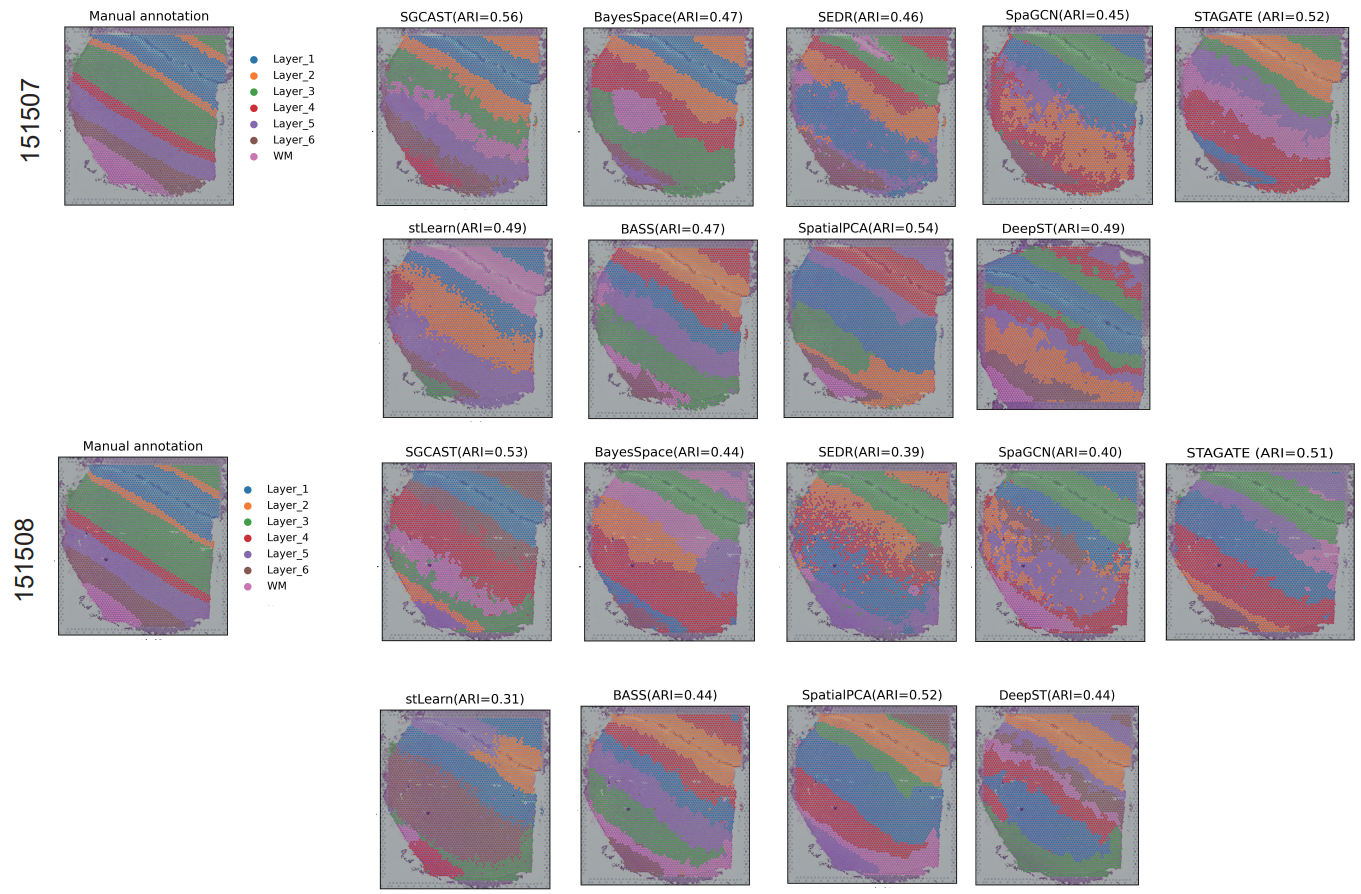


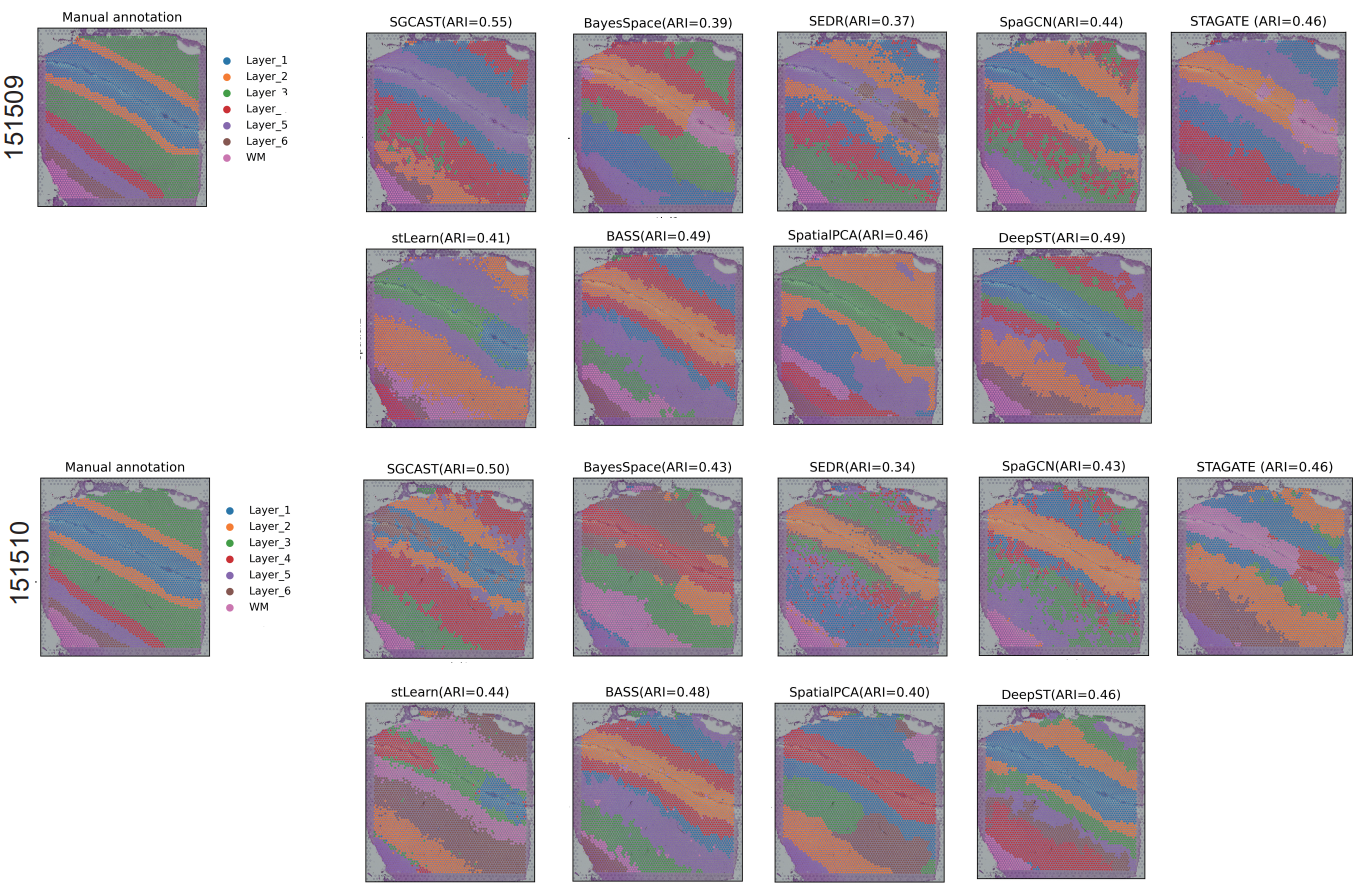


**Supplementary Fig 1.** The comparison of clustering results by SGCAST, BayesSpace, SEDR, SpaGCN, STAGATE, stLearn, BASS, SpacialPCA, DeepST, and manual annotation in slides 151507, 151508, 151509, and 151510 of the DLPFC dataset.


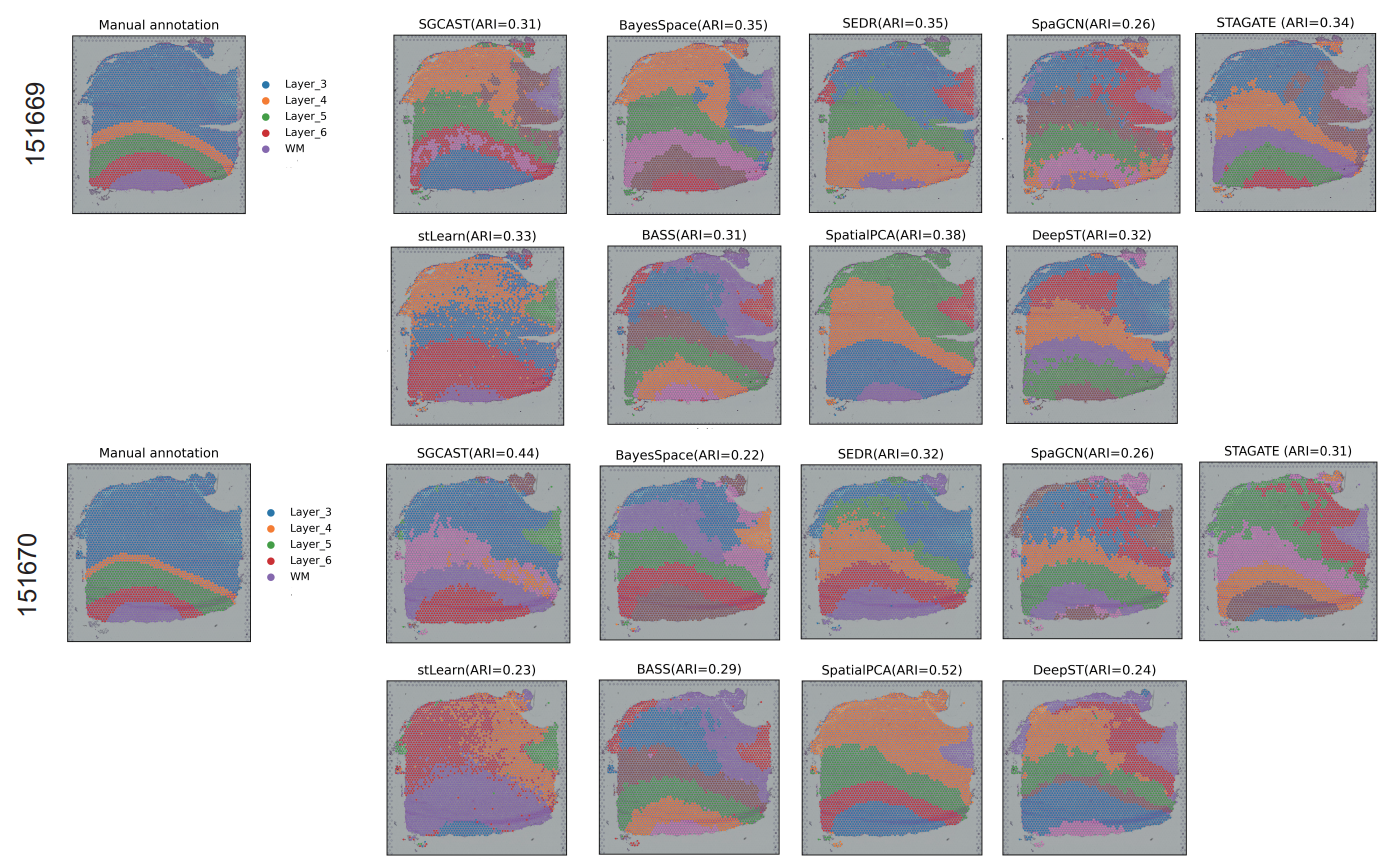

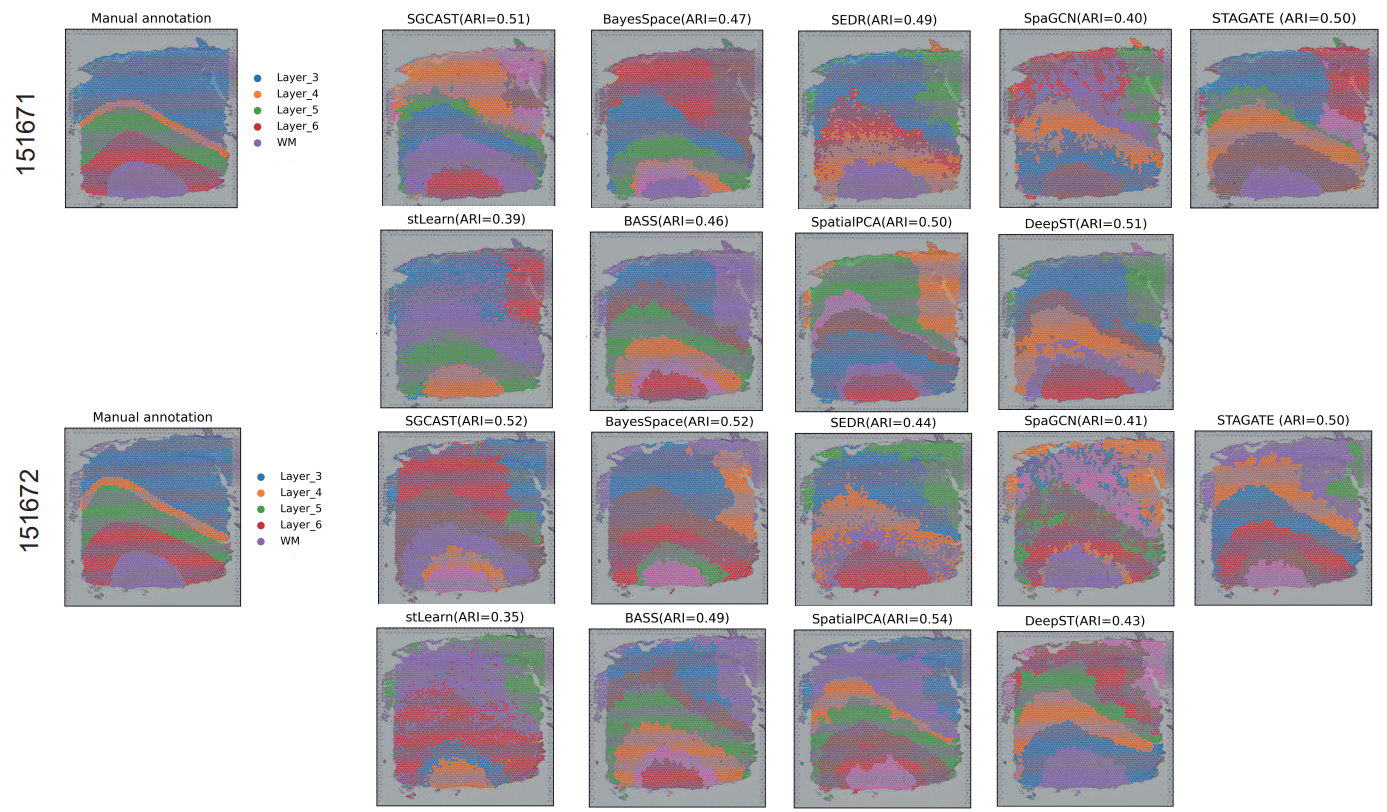


**Supplementary Fig 2.** The comparison of clustering results by SGCAST, BayesSpace, SEDR, SpaGCN, STAGATE, stLearn, BASS, SpacialPCA, DeepST, and manual annotation in slides 151669, 151670, 151671, and 151672 of the DLPFC dataset.


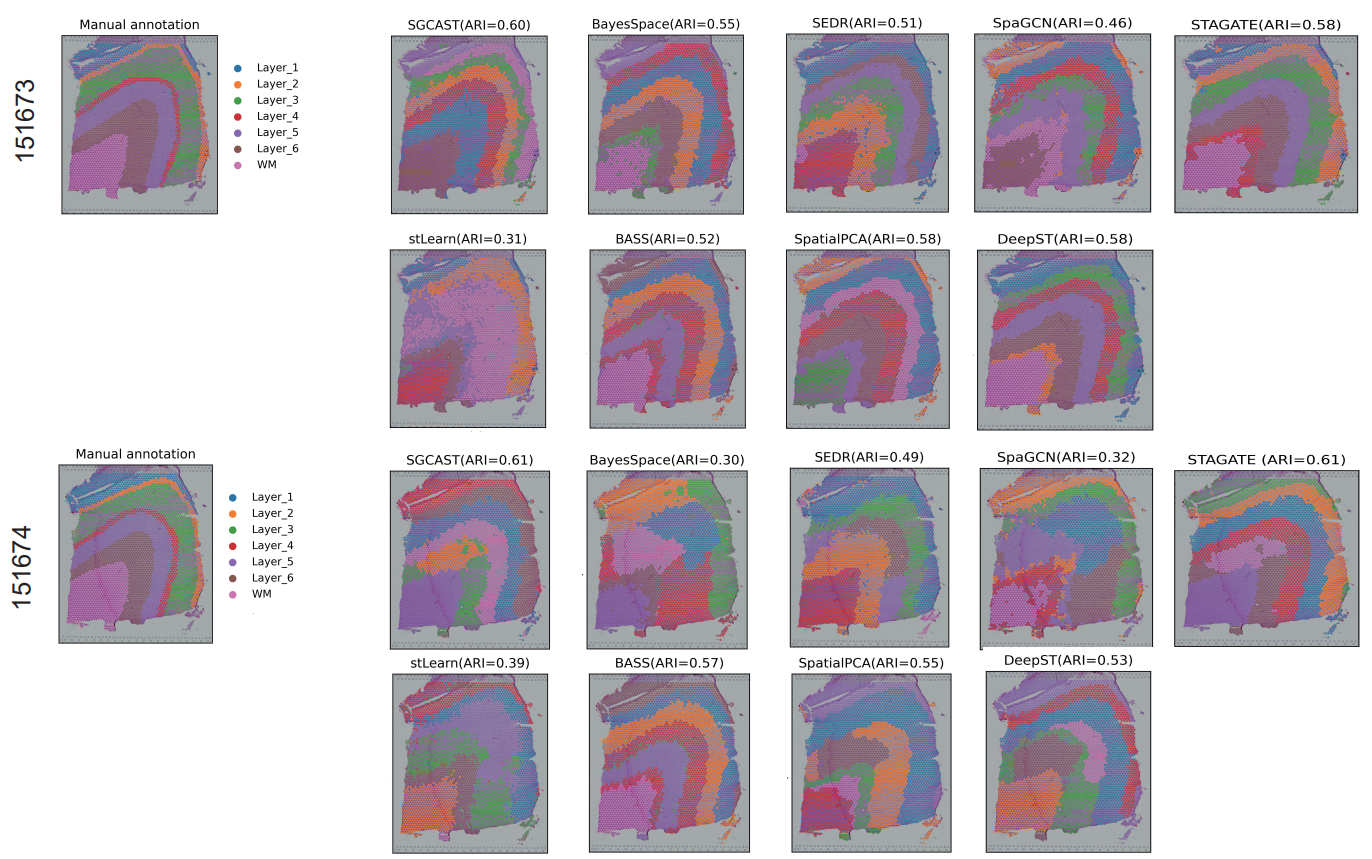

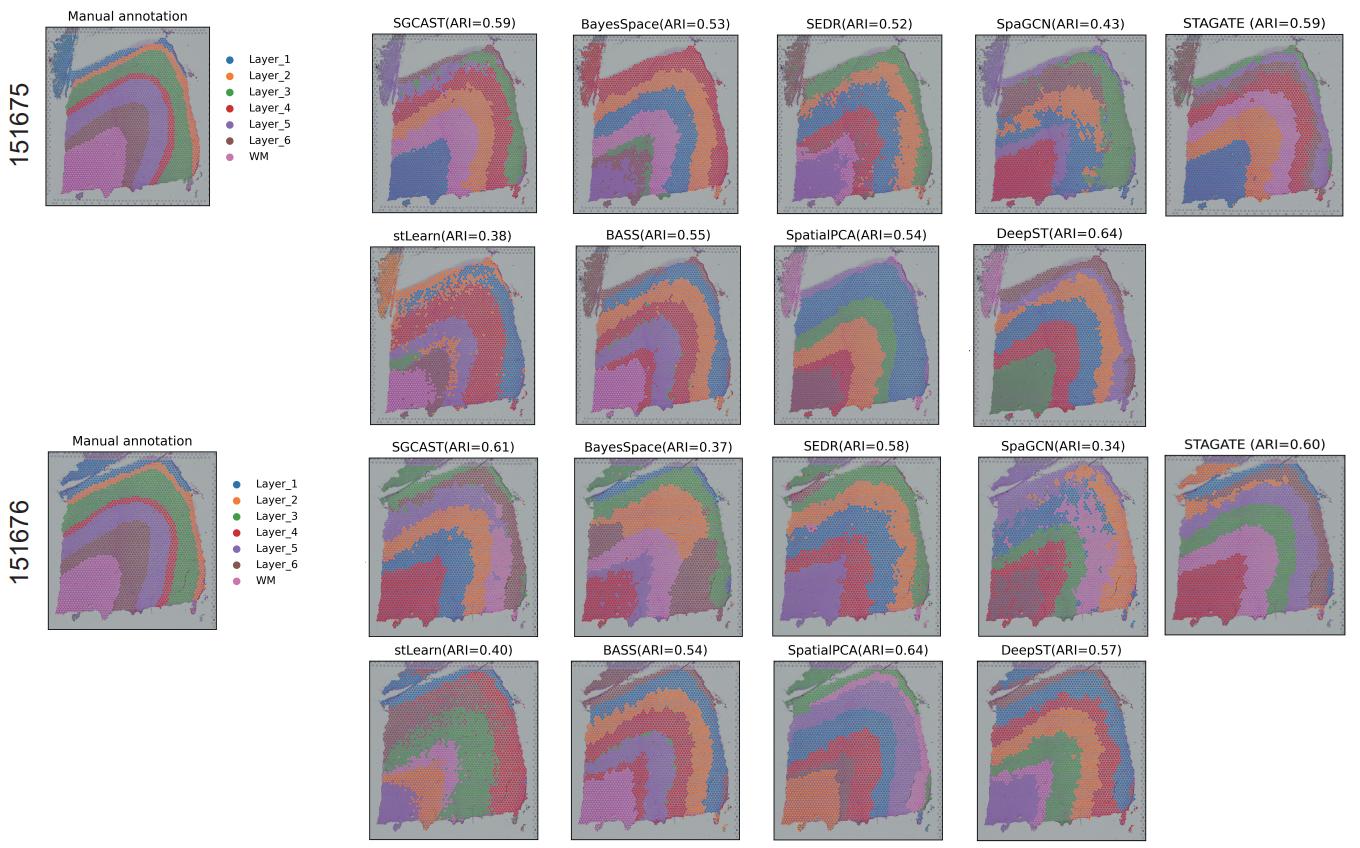


**Supplementary Fig 3.** The comparison of clustering results by SGCAST, BayesSpace, SEDR, SpaGCN, STAGATE, stLearn, BASS, SpacialPCA, DeepST, and manual annotation in slides 151673, 151674, 151675, and 151676 of the DLPFC dataset.


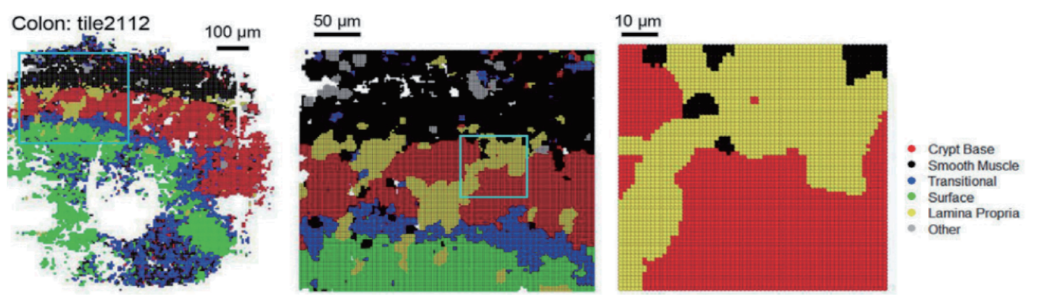


**Supplementary Fig 4.** Annotation of the Seq-scope Colon [4].


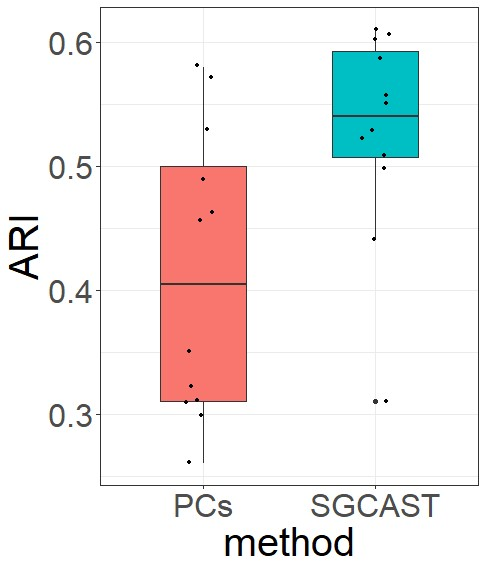


**Supplementary Fig 5.** Boxplot of ARIs for applying mclust directly to PCs vs latent embeddings learned by SGCAST.


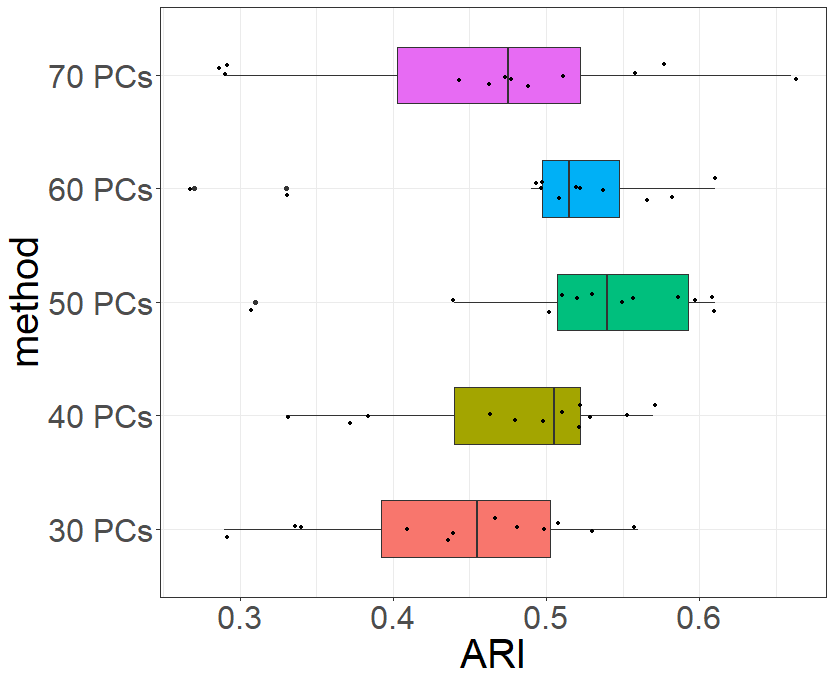


**Supplementary Fig 6.** Boxplot of ARIs for different numbers of top PCs employed by SGCAST on DLPFC dataset.


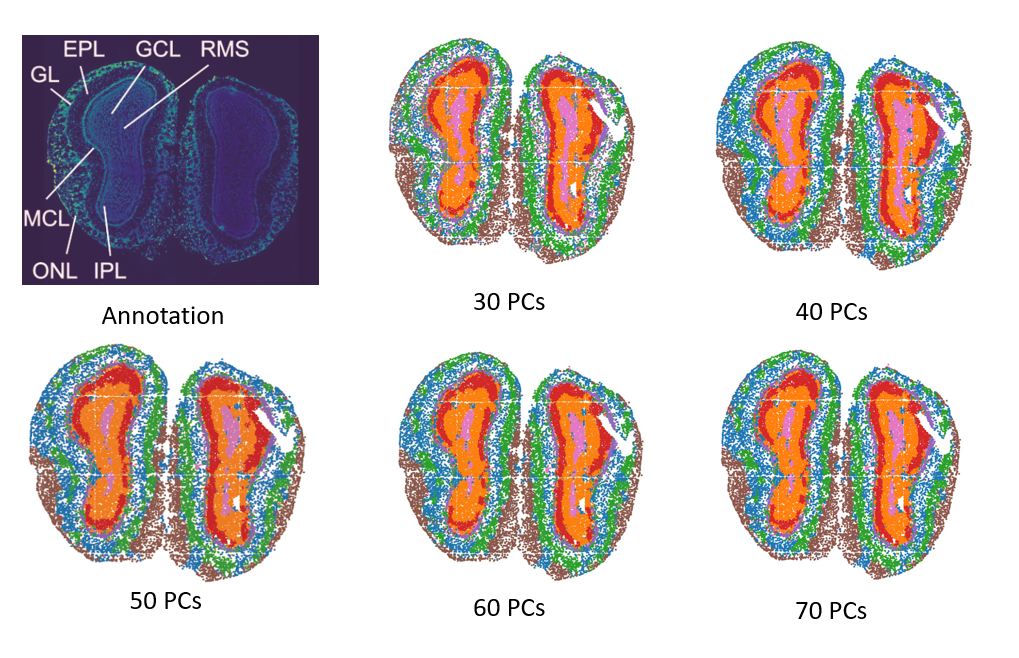
**Supplementary Fig 7.** The annotation and results of SGCAST using different number of PCs on Stereo-seq mouse olfactory.


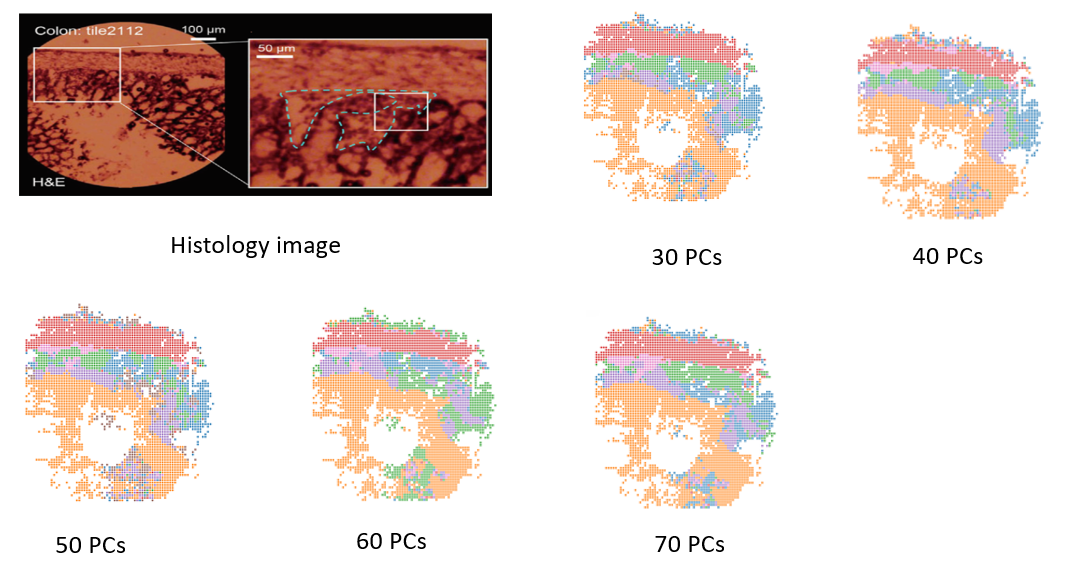


**Supplementary Fig 8.** The histology image and results of SGCAST using different number of PCs on Seq-scope mouse colon.


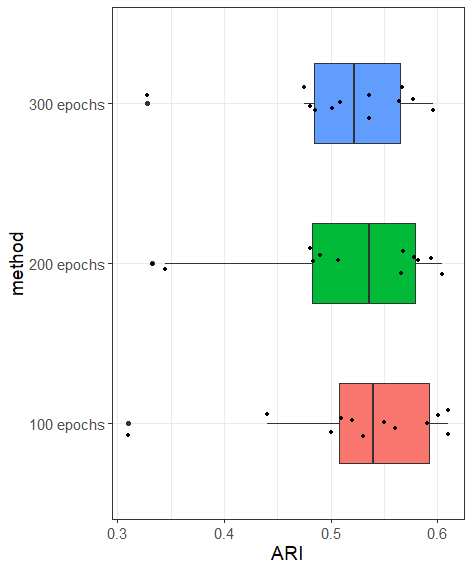


**Supplementary Fig 9.** Boxplot of ARIs for different epoch numbers used by SGCAST on DLPFC dataset.


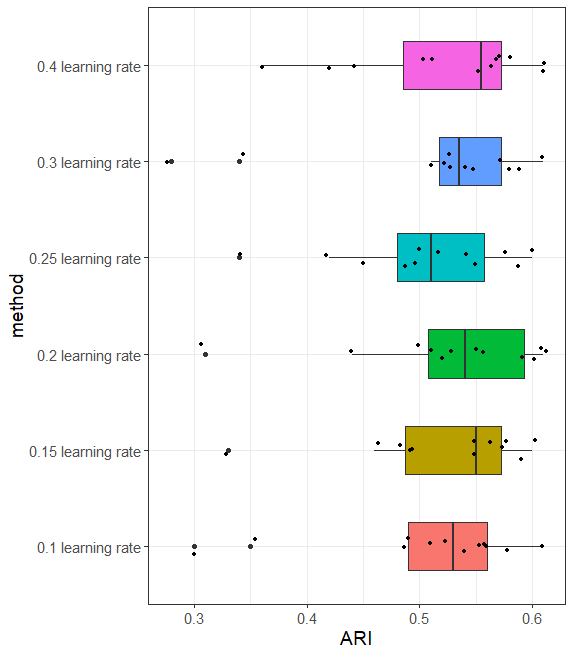


**Supplementary Fig 10.** Boxplot of ARIs for different learning rates used by SGCAST on DLPFC dataset.


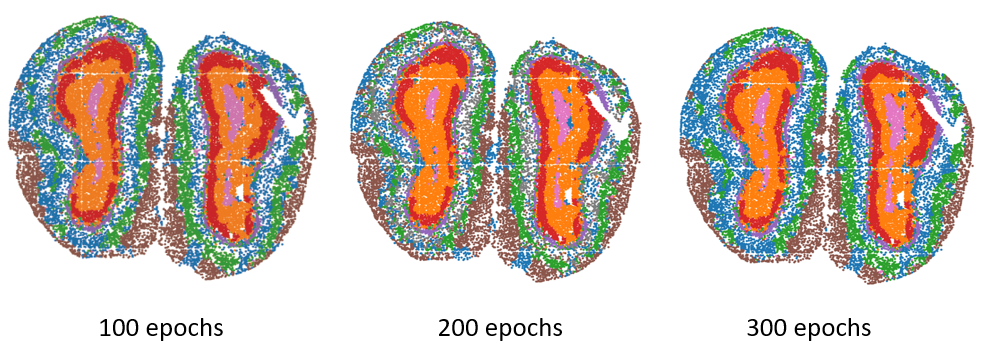


**Supplementary Fig 11.** The results of SGCAST using different number of epochs on Stereo-seq mouse olfactory.


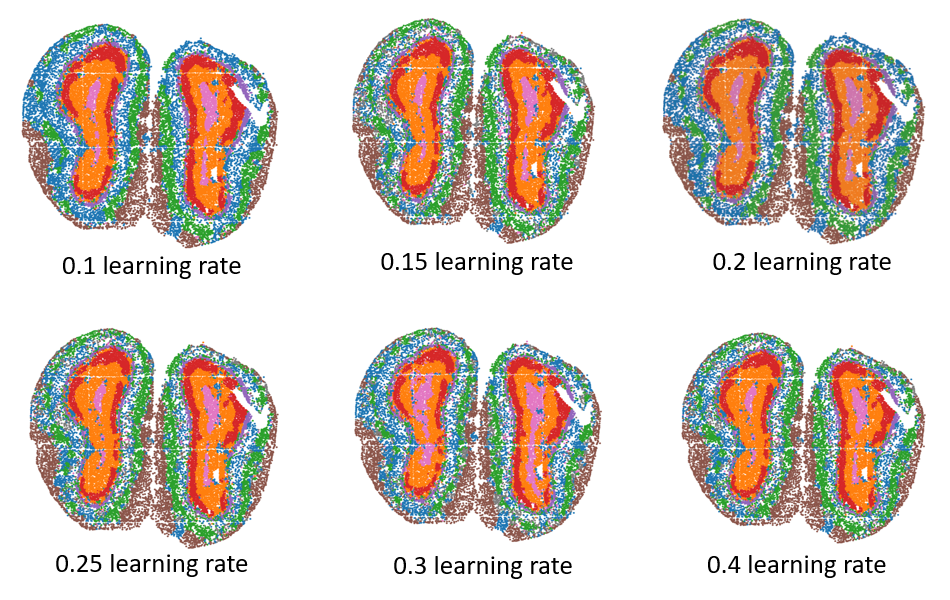


**Supplementary Fig 12.** The results of SGCAST using different learning rates on Stereo-seq mouse olfactory.


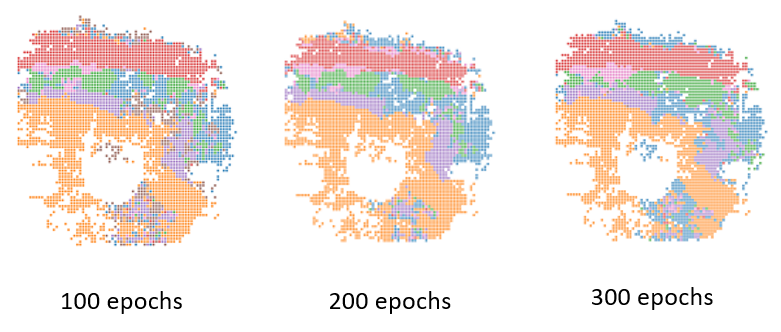
 **Supplementary Fig 13.** The results of SGCAST using different number of epochs on Seq-scope mouse colon.


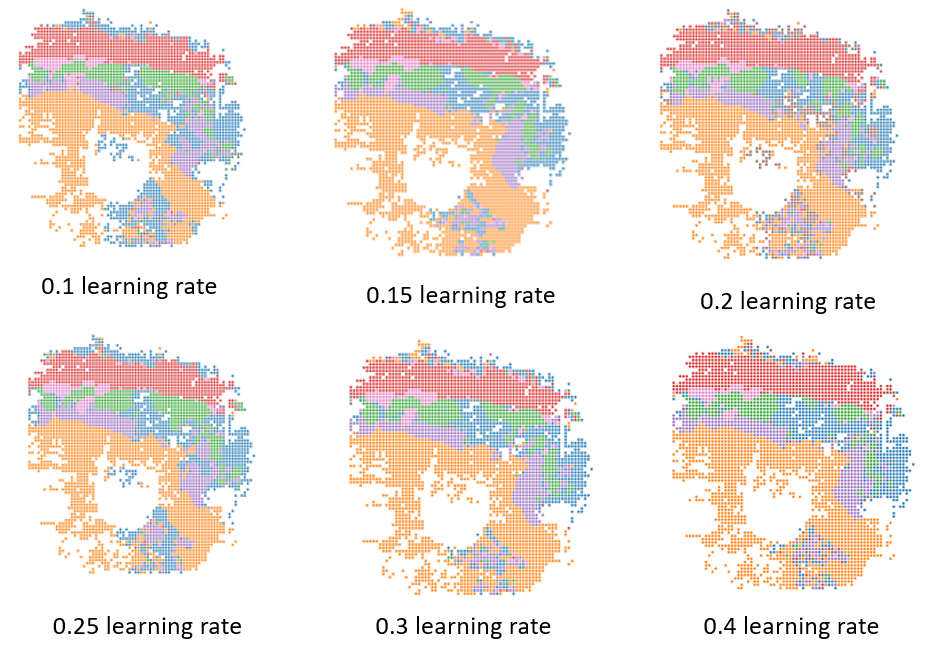


**Supplementary Fig 14.** The results of SGCAST using different learning rates on Seq-scope mouse colon.

| Dataset | Platform | Spots number |
| --- | --- | --- |
| DLPFC 151673 | 10X Visium | 3639 |
| DLPFC 151671 | 10X Visium | 4110 |
| DLPFC 151509 | 10X Visium | 4789 |
| Mouse olfactory bulb | Stereo-seq | 19109 |
| Mouse colon | Seq-scope | 28399 |
| Embryo12.5 | Stereo-seq | 51335 |
| Embryo14.5 | Stereo-seq | 102489 |
| Embryo16.5 | Stereo-seq | 121764 |

**Supplementary Table 1.** Details of datasets.

# Settings of other spatial clustering methods:

# BayesSpace: BayesSpace was applied to the DLPFC datasets. The input is the top 15 PCs of the log-normalized expression of the top 2,000 HVGs. In *spatialCluster()*, *nrep* was set as 50,000 and *gamma* was set to 3.

# stLearn: stLearn was applied to the DLPFC dataset. First, stLearn.spatial.SME.SME_normalized() function was conducted on the raw gene by spot matrix with the parameter *use_data*= “raw” and *weights*=” physical_distance”. Then top 30 PCs were used for clustering.

# SpaGCN v1.2.5: SpaGCN was applied to the DLPFC dataset, Seq-scope colon, and Stereo-seq mouse olfactory bulb. For the DLPFC dataset, we set parameters following its online tutorial. For the rest two dataset, we calculate the adjacency matrix using spg.calculate_adj_matrix with the parameter *a* = 1, *b* = 49, *histology* = False; the resolution for Louvain: *res* = 1.0.

# SEDR: SEDR was applied to the DLPFC dataset, Seq-scope colon, and Stereo-seq mouse olfactory bulb. For the DLPFC dataset, we conducted SEDR following its online tutorial with *k* = 10, and the number of epochs is 200. For the rest two dataset, the *resolution* for Leiden is set to 0.8.

# BASS: BASS was applied to the DLPFC dataset, Seq-scope colon, Stereo-seq mouse olfactory bulb and E12.5. For the DLPFC dataset, we set parameters following its online tutorial. For the Seq-scope colon, and Stereo-seq mouse olfactory bulb, *C* = 20, *R* = 7. For E12.5, *C* = 20, *R* = 25.

# SpacialPCA: SpacialPCA was applied to the DLPFC dataset, Seq-scope colon, Stereo-seq mouse olfactory bulb and E12.5. For the DLPFC dataset, we set parameters following its online tutorial. For the rest dataset, select spatial genes using sparkx. For the Seq-scope colon, and Stereo-seq mouse olfactory bulb, *cluster_numbers* = 7. For E12.5, *cluster_numbers* = 25.

# STAGATE: STAGATE was applied to the DLPFC dataset, Seq-scope colon, Stereo-seq mouse olfactory bulb and embryo datasets. For the DLPFC and Stereo-seq mouse olfactory bulb, we set parameters following its online tutorial. For the Seq-scope colon, and Stereo-seq embryo datasets, *rad_cutoff* = 50 and the resolution for Louvain: *res* = 0.8.

# DeepST: DeepST was applied to the DLPFC dataset. For the DLPFC dataset, we set parameters following its online tutorial.

# Relationship between adjacency matrices in Encoder and decoder of SGCAST:


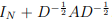
Following Kipf and Welling [1], the adjacency matrix in the graph convolutional layer has the form, where *I_N_* is the identity matrix, *D* is the degree matrix and *A* is the affinity matrix. *D* tells the number of neighbors for each vertex in a graph. *A* describes connectivity in a graph with entries 0 or 1. Based on the form, the adjacency matrix is symmetric with all diagonal elements equal to 1 while all non-diagonal elements are non-negative and less than 1. Note that entries in the adjacency matrix *W_i_* calculated in the encoder of SGCAST are in the same range as above.

Because the encoder performs Laplacian smoothing that makes the latent representation of each node similar to those of its neighboring nodes, on the contrast, the decoder should reverse the latent embedding back into reconstructed principal components [2]. Following GALA [3], the reconstruction of node features in the decoder is designed based on Laplacian sharpening as the counterpart of Laplacian smoothing, where the adjacency matrix in the graph convolutional layer has the form
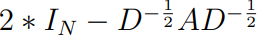
.The sum of the adjacency matrix in Laplacian smoothing and sharpening is *3* I_N_*. As a result, the adjacency matrix $\hat{W}$*_i_* in the decoder layer has the form *3*I_N_* - *W_i_*.

# Difference between SGCAST and SEDR:

While both methods use auto-encoders to learn gene expression and spatial embedding information, there are several key differences between the two approaches.

First, the structure of the auto-encoder is fundamentally different. SEDR uses one deep auto-encoder for gene expression and one variational graph auto-encoder for spatial embedding, and concatenates the learned features. In contrast, SGCAST uses a symmetric graph convolutional auto-encoder to aggregate the information, rather than concatenating it. This approach allows SGCAST to better capture the spatial information and improve the clustering performance.

Second, SEDR uses an adjacency matrix with entries equal to 0 or 1 to describe the neighbourhood, which can lead to issues when working with large datasets. In contrast, the adjacency matrix used in the GCN layer of SGCAST captures the relative closeness between spots in the batch, and does not require the construction of a full adjacency matrix for all spots.

Finally, the decoder part of the auto-encoder in SEDR uses one fully connected layer to reconstruct the expression matrix from concatenated gene embedding and spatial embedding, while the decoder in SGCAST reverses the Laplacian smoothing in the encoder to do Laplacian sharpening, which provides a more intuitive interpretation of the learned features.

# Hardware: The training of GPU methods was conducted on a Tesla V100 GPU.

# References

1. Kipf TN, Welling M. Semi-supervised classification with graph convolutional networks. In *Proc. International Conference on Learning Representations*, 2016.
2. Li Q, Han Z, Wu XM. Deeper insights into graph convolutional networks for semi-supervised learning. In *Thirty-Second AAAI conference on artificial intelligence*, 2018.
3. Park J, Lee M, Chang HJ, et al. Symmetric graph convolutional auto-encoder for unsupervised graph representation learning. In *Proceedings of the IEEE/CVF International Conference on Computer Vision*, 2019; 6519-6528.
4. Cho CS, Xi J, Si Y, et al. Microscopic examination of spatial transcriptome using Seq-Scope. *Cell* 2021; 184(13): 3559-3572.
